# Persistent occiput posterior position and stress distribution in levator ani muscle during vaginal delivery computed by a finite element model

**DOI:** 10.1007/s00192-019-03997-8

**Published:** 2019-06-13

**Authors:** Linda Havelková, Ladislav Krofta, Petra Kochová, Václav Liška, Vladimír Kališ, Jaroslav Feyereisl

**Affiliations:** 1 New Technologies-Research Centre, University of West Bohemia, Univerzitní 22, Plzeň, Czech Republic; 2 Institute for the Care of Mother and Child, Podolské nábřeží 157, 14700 Praha, Czech Republic; 3 3rd Medical Faculty, Charles University, Ruska 2411/87, Praha, Czech Republic; 4 European Centre of Excellence, Faculty of Applied Sciences, University of West Bohemia, Univerzitní 22, Plzeň, Czech Republic; 5 Faculty of Medicine in Pilsen, Charles University, Lidická 1, Plzeň, Czech Republic; 6 Biomedical Center, Faculty of Medicine in Pilsen, Charles University, Praha, Czech Republic; 7 Department of Obstetrics and Gynecology, University Hospital, Plzeň, Czech Republic

**Keywords:** FEM modeling, Levator ani muscle trauma, Ogden material model, Persistent occiput posterior position, Vaginal delivery

## Abstract

**Introduction and hypothesis:**

Objective of this study was to develop an MRI-based finite element model and simulate a childbirth considering the fetal head position in a persistent occiput posterior position.

**Methods:**

The model involves the pelvis, fetal head and soft tissues including the levator ani and obturator muscles simulated by the hyperelastic nonlinear Ogden material model. The uniaxial test was measured using pig samples of the levator to determine the material constants. Vaginal deliveries considering two positions of the fetal head were simulated: persistent occiput posterior position and uncomplicated occiput anterior position. The von Mises stress distribution was analyzed.

**Results:**

The material constants of the hyperelastic Ogden model were measured for the samples of pig levator ani. The mean values of Ogden parameters were calculated as: μ1 = 8.2 ± 8.9 GPa; μ2 = 21.6 ± 17.3 GPa; α1 = 0.1803 ± 0.1299; α2 = 15.112 ± 3.1704. The results show the significant increase of the von Mises stress in the levator muscle for the case of a persistent occiput posterior position. For the optimal head position, the maximum stress was found in the anteromedial levator portion at station +8 (mean: 44.53 MPa). For the persistent occiput posterior position, the maximum was detected in the distal posteromedial levator portion at station +6 (mean: 120.28 MPa).

**Conclusions:**

The fetal head position during vaginal delivery significantly affects the stress distribution in the levator muscle. Considering the persistent occiput posterior position, the stress increases evenly 3.6 times compared with the optimal head position.

## Introduction

In the past decades, the concept of pelvic floor trauma was attributed largely to perineal, vaginal and anal sphincter injuries. In recent years with advances in magnetic resonance imaging (MRI) and four-dimensional sonography, it has also become evident that levator ani muscle (LAM) injuries represent an important part of pelvic floor trauma [1, 2]. LAM injuries occur in 13–36% of women who have vaginal delivery (VD), and there is conclusive evidence of a connection between these injuries and pelvic floor dysfunction (PFD) [3, 4]. During VD, the LAM undergoes extremely large deformations to allow the passage of the fetal head [5]. These deformations can lead to injuries such as muscle tearing and striated muscle atrophy, owing to pudendal denervation [6]. This trauma usually causes lifelong complications [7].

A persistent occiput posterior (OP) position in the second stage of delivery carries an increased risk of labor complications, obstetric interventions, severe maternal perineal lacerations and anal sphincter injury [8, 9]. This condition is found in up to 5% of deliveries [10] and occurs significantly more often in first labors.

Several virtual models of VD have already been developed. Most of them are focused on the second stage of labor, starting from full dilatation of the cervix to the birth of the fetus [11–14]. Although these models provide satisfactory insights, they are strongly limited by constitutive data or boundary conditions representing the real anatomy and physiology. Material properties are usually derived from cadaveric LAM uniaxial or biaxial tests [15]. To the authors' knowledge, there are no studies describing the material properties of non-cadaveric LAM. The objective of this study was to develop a sophisticated MRI-based finite element model of the female pelvic floor to simulate a child birth for fetal head position in persistent OP and analyze the stress distribution in the LAM with respect to the cardinal movements of labor.

## Materials and methods

The study was approved by the local ethics committee of the authors' institution. An MRI-based three-dimensional computer model of the bony pelvis, pelvic floor muscles, pelvic floor organs and fetal head was created. All supporting structures were replaced by boundary conditions. The geometry of these structures was based on live-subject MRI data. Female characteristics were 25 years old, BMI 21.9 kg/m^2^, nulliparous, Caucasian, no previous vaginal delivery, normal POP-Q points, and absence of PFD symptoms. The neonate was 1 day old, after uncomplicated VD at term, with neurological indications for an MRI brain scan. The volunteer/legal representative gave written consent. Our method of obtaining the three-dimensional computer model and the imaging protocol have been published previously [16]. The objective of this study was to simulate changes in the LAM during vaginal delivery with respect to the cardinal movements of labor.

The original MRI-based 3D finite element model of the female pelvic floor already developed by these authors was improved and used for further applications. Compared with the original model, the following changes were implemented: (1) the real, not simplified geometry of the obturator internus muscle (OIm) was involved, (2) the LAM was clearly split into three individual parts, the iliococcygeal muscle (ICm), puborectal muscle (PRm), and pubovisceral muscle (PVm), based on in-vivo MRI, (3) the real passive biomechanical properties of pelvic floor muscles obtained from measurements were newly used, (4) the boundary conditions representing the pelvic floor-supporting apparatus were particularized in detail, and (5) OP fetal head positions were considered.

### Model geometry and finite element mesh (FEM)

The initial model geometry was reconstructed from in vivo scanned MRI using a free semiautomatic software (3D Slicer, 3.0; BWH, Boston, MA, USA). The outlines of relevant structures were digitized from these consecutive axial MRI scans. The resulting geometry and mesh were created in HyperMesh commercial software (11.0; Altair, MI, USA). Rigid parts, such as the female pelvis and fetal head, were constructed with 2D triangular mesh including 119,373 elements in summary. Deformable parts, such as muscles, were modeled by 3D tetrahedral mesh consisting of 527,164 elements.

The accuracy and efficiency of the finite element simulations considered in the presented work are highly predisposed to the quality of the finite element mesh [17]. Thus, the process of element quality control was not neglected. The tetrahedral elements (four-node version) were chosen to discretize the model geometry. The aspect ratio (AR), measured as the ratio of the longest edge length divided by the minimum altitude of the smallest side, represents a simple parameter quantifying the element shapes. Prior models recommended that the percentage of the tetrahedral element ARs > 3 should be < 5% to produce the smallest errors [18, 19]. In this work, 1.19% of element ARs > 3 was considered. In addition, respecting the published data [20], only dihedral angles between 30°and 120° were accepted in this study. The size of the element edges was also limited; the range of 1–3 mm was considered. Finally, the element Jacobians were also checked. This parameter represents the determinant of the Jacobian matrix containing information regarding the volume, shape and orientation of the element. The following criteria were strictly used: (1) a positive value [19]; (2) > 0.2 in magnitude [18, 21]; (3) < 5% of all Jacobians below a magnitude of 0.7 [22].

### Material properties

During vaginal delivery, the LAM undergoes extremely large deformation; therefore, the hyperelastic nonlinear Ogden material model was used [23]. The Ogden material model is a mathematical model developed by Raymond Ogden in 1972. This model describes hyperelastic models having non-linear stress-strain behavior. The model is suitable for complex materials such as biological tissues because its material characteristic is very similar. In our work, the human cadaveric muscle samples were replaced by animal tissues removed in vivo. The material parameters were based on experimental measurements of porcine muscle samples.

#### Experimental procedure—porcine LAM specimen

The experiments conducted were in accordance with the animal welfare regulations and guidelines for the country in which the experiments were performed. Twenty LAM samples from ten female pigs (age: 12–14 weeks, weight: 33–45 kg, nulliparous) were obtained from practice surgical operations designed for medical students. The LAM samples were surgically removed during general anesthesia. Two slabs of LAM were obtained from each pig (from the right and left side). After that, the LAM specimens were individually stored in glass laboratory jars and immediately deep frozen (−20 °C) until further processing. The effects of careful deep freezing on the hyperelastic and tensile properties are negligible, as reported in the literature [24].

#### Experimental measurement—passive biomechanical LAM properties to determine the stress-strain curve

The frozen LAM slabs were kept at room temperature (22 °C) to thaw out for 3 h before mechanical measurement following the recommended procedure [24]. The muscles were cleared of fascia and cut into cuboid-shaped samples suitable for the measurement device (about 6 mm wide, 4 mm thick and 10 mm long), always in the direction of the muscle fibers. The fibers were always identified anteriorly to the ischial spine, posteriorly to the pubic rami, medially to the arcus tendineus musculi levatoris ani (ATML) and laterally to the vaginal wall. The LAM specimens were clamped in a mechanical test system in the direction of muscle fibers, and a uniaxial mechanical test was performed. The Zwick/Roell Z050 traction machine (Zwick/Roell, Ulm, Germany) was used for measurement. The exact width and thickness of the initial unloaded samples were measured by a digital caliper to obtain the initial cross-sectional areas. The lengths of unloaded samples were given by the initial distance between the grips of the measuring apparatus. Thus, the original length of each specimen was found to be 5 or 10 mm. The measuring apparatus does not allow keeping the samples in liquid during the measurement. Therefore, they were sprayed with 0.9% NaCl (sodium chloride) to prevent dehydration. Each sample underwent preconditioning consisting of 20 cycles up to 15% of its original length. The uniaxial extension was performed until the muscle ruptured. The loading velocity was 6 mm/min, as reported in [25]. The tensile force and sample elongations were recorded.

#### Experimental procedure—Ogden material identification

The simple homogeneous deformations were considered during uniaxial tension: x_i_ = λ_i_ X_i_, where i = 1, 2, 3 and x_i_ are the corresponding coordinates after deformations, λ_i_ are constants referred to as the principal stretches of deformations, and X_i_ are coordinates identifying material particles in some unstressed configuration. The deformation gradient, **F**, in terms of principal stretches is then: **F** = diag [λ_1_, λ_2_, λ_3_]. For an incompressible material, the principal stretches satisfy the constant: λ_1_ λ_2_ λ_3_ = 1. Thus, for the Ogden hyperelastic material, the strain function is given by the following formula:

$$ W=\sum \limits_{i=1}^3\sum \limits_{j=1}^N2\frac{\mu_j}{\alpha_j}\left({\left(\det {\boldsymbol{F}}^{-\frac{1}{3}}{\lambda}_i\right)}^{\alpha_j}-1\right)+\frac{K}{2}{\left(\det \boldsymbol{F}-1\right)}^2, $$

where μ_j_ [GPa] and α_j_ [−] are the unknown Ogden parameters and N is the number of terms in the Ogden series; for the purpose of this work, *N* = 2. Assuming uniaxial tension, the unknown variables are then: λ_2_ = λ_3_ = λ_1_^–1/2^, J = 1. Thus, the parameter K is absolutely not significant for this purpose. Moreover, it is assumed that the muscular tissue exhibits an incompressible behavior. The measured tensile force and elongation were used to calculate the stress-strain curve for each muscle sample. The stress was defined as the actual force divided by the initial cross section. The strain was defined as the actual specimen elongation divided by the initial specimen length. The final stress-strain curves were used to identify unknown parameters in the specific strain-energy function by means of the least squares technique. For the case of uniaxial tension, the nominal stress-stretch relation for Ogden material can be written as: $\mathrm{P}=\frac{\updelta \mathrm{W}}{\updelta \uplambda}$. The method consists of minimizing the stress error and thus:

$$ E=\frac{1}{2}\sum \limits_{i=1}^{ND}{\left({P}_i^{test}-{P}_i^{derived}\right)}^2, $$

where P_i_^test^ is the measured nominal stress for the nominal strain measure, P_i_^derived^ is the stress obtained from the strain energy function, and ND is the number of data points. In this study, the function lsqcurvefit in the Optimization Toolbox of MATLAB (R2013a; The MathWorks, Inc., Natick, MA, USA) was used. The lower and upper limits were added to ensure the following inequality that must be satisfied for all Ogden parameters: $\sum \limits_{\mathrm{i}=1}^{\mathrm{N}}{\upmu}_{\mathrm{i}}{\upalpha}_{\mathrm{i}}>0$ [16].

The density of general mammalian skeletal muscle tissues is approximately 1.06 kg/l [26]; the value of Poisson’s ratio is 0.499, as usual. The friction between the fetal head and female pelvic floor muscles is 0.1 assuming that the contact is well lubricated during the vaginal delivery.

### Initial and boundary conditions

The initial (ICs) and boundary (BCs) conditions were considered to simulate the real behavior of pelvic structures during VD. The ICs reflect the initial positions and rotations of all observed components. The BCs represent the surrounding support of all model structures. The pelvis was modeled by a rigid body fixed for all degrees of freedom in 3D space and represents the mechanical frame for soft tissues. In addition, it forms the anatomical pelvis axis limiting the head trajectory during labor. Passive connective tissue in the model followed the outlines of relevant structures. The obturator membrane was replaced by external supporting forces. The OIm also provides support for the LAM and was fixed to the bony pelvis (ischiopubic ramus) and at the points where the OIm leaves the internal pelvic floor in the direction of muscle insertion—the medial aspect of the greater trochanter. The ATML traverses the anteromedial aspect of the OIm. To create the LAM model, three sections of levator ani muscles, based on existing descriptions, were included [27]. The ICm originates from the ATML. These nodes were fixed to the OIm. Laterally, the ICm is attached to the posterior ilia and dorsal to the sacrum; this part of the muscle is also translationally and rotationally fixed. Nodes along the upper cranial third of the ICm were limited to move only in the plane defined by the sacrum and posterior ilia in the interior and lateral directions. This condition represents the LAM support provided by ligaments and tendons attached to the inner pelvic walls. The PVm and PRm originate from the fibrous enthesis on the dorsal surface of the pubic bone. These muscle parts are fixed in the space. The upper arc of the PVm is connected to the ICm; the lower part of the PRm is then connected to the PVm. All other nodes of the presented model were unconstrained.

**Fig. 1:**
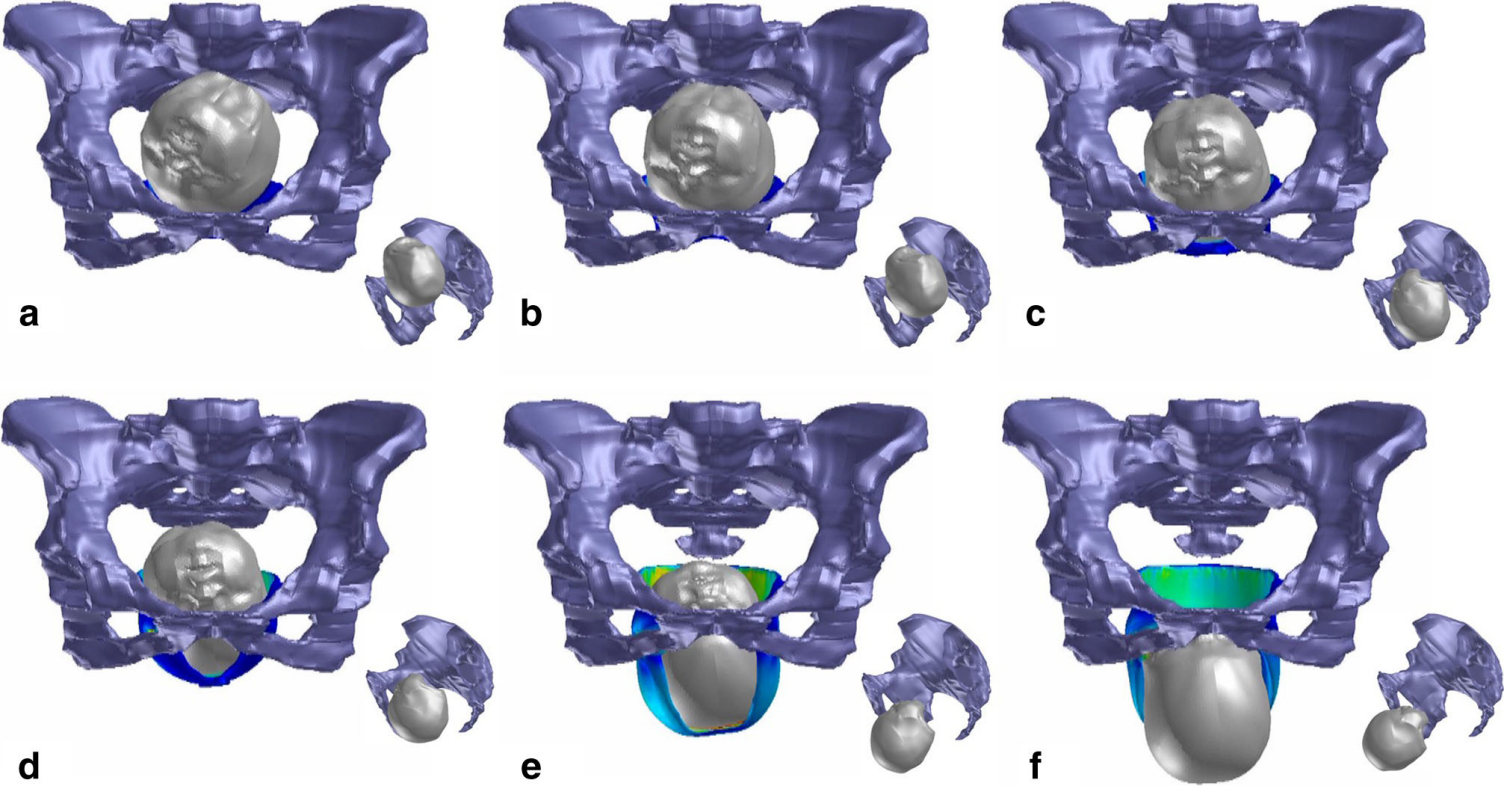
Sequence of six images showing the simulated effects of fetal head descent and internal rotation in the second stage of labor in OP. At the level of the pelvic inlet, the fetal head is in the LOP position. **a** The head is engaged in the pelvic inlet. The leading point is localized 3 cm over line 0 (*station −3*). **b** The leading point is localized 1 cm over the ischial spine and 1 cm over line 0 (*station −1*). **c** The leading point is localized 3 cm below line 0 (*station +3*). The internal rotation is completed when the leading point is localized below the midpelvic plane. **d** Further fetal head descent; the leading point is localized 5 cm below line 0 (*station +5*). **e** and **f** After the ischial spine has been passed and the forehead has completely passed the pubic bone, the flexion of the head becomes possible and abruptly twists up toward the outlet

**Fig. 2:**
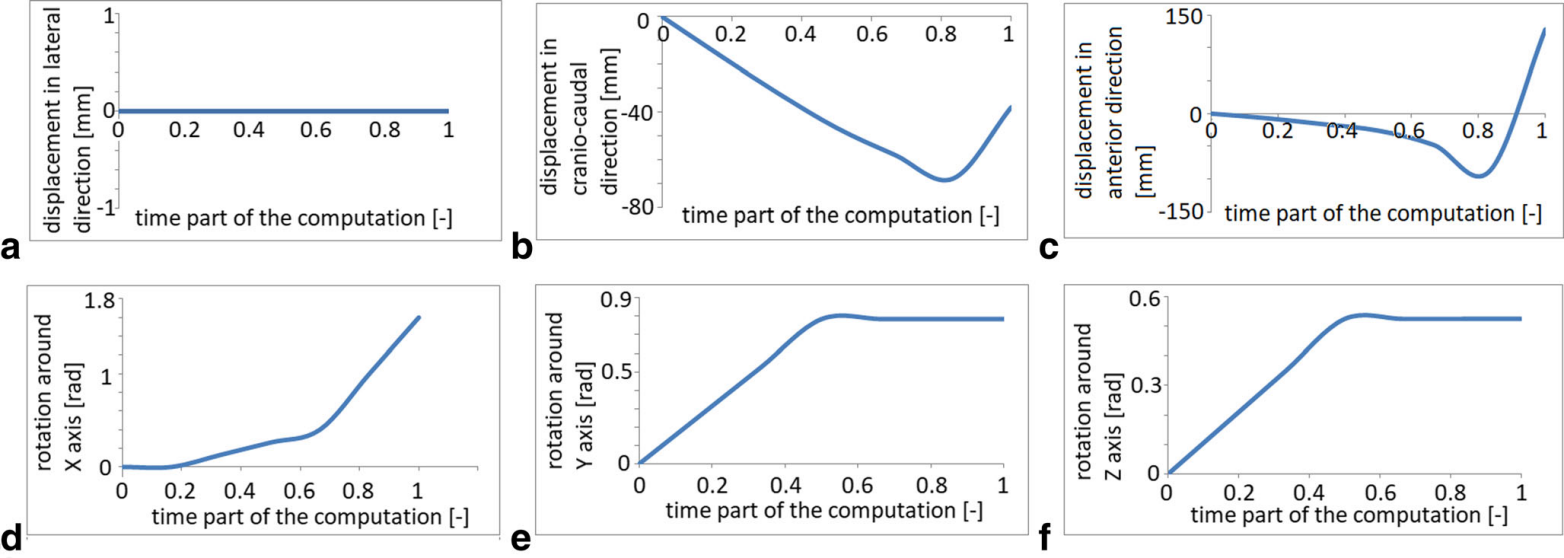
Biomechanical description of the fetal head trajectories in the birth canal (displacement, internal rotation, extension) for the case of OP. During the internal rotation, there was no lateral shift to the left or right side (**a**). In the craniocaudal direction, the head descends, maintaining a downward direction until the forehead passes the pubic bone and then twists up toward the outlet (**b**). The displacement in the anterior direction from the beginning to the end of the simulation is 100.0 mm (**c**). In the midpelvis during the internal rotation, the head undergoes 20° flexion. After passing the ischial spine, the flexion increases toward the outlet (**d**). The head, which initially deviates 45° degrees, straightens (**e**). The fetal head model enters the pelvis in the left OP position. The internal rotation is completed at station +3. The posteriorly positioned occiput rotates in the midpelvis to the os sacrum through 45° (**f**)

The fetal head was also replaced by a rigid body without any possibility to be deformed [16]. For station, following obstetric convention, the pelvis was divided above and below the ischial spine into levels (expressed in centimeters). There is a difference of head descent when the occiput is posterior. Fetuses with occiput anterior (OA) position follow the curved path of the birth canal, and the fetal head undergoes positional changes through the birth canal (cardinal movements). In this study, the fetal head entered the pelvic inlet in the left occiput posterior (LOP) position, and the internal rotation moved the occiput toward the os sacrum. At the level of the ischial spine (station 0), the internal rotation was completed and the occiput could remain in the direct occiput posterior position. After the ischial spine has been passed and the forehead has completely passed the pubic bone, flexion of the head becomes possible, and it abruptly twists up toward the outlet (Fig. 1). A biomechanical description of the fetal OP head trajectory in the birth canal is shown in Fig. 2.

### Stress distribution in the MLA

A vaginal delivery scaled in seconds was simulated for the head position in OP. At the beginning of the simulation, the LAM remained at rest without any external load. The distribution of von Mises stress generated in the LAM during vaginal delivery was analyzed using the FEM method and the Virtual Performance Solution commercial software (VPS 9.0; ESI Group, Paris, France). The von Mises stress allows any arbitrary three-dimensional stress to be represented as a single positive stress value. All three principal stresses could be computed and shown.

## Results

**Fig. 3:**
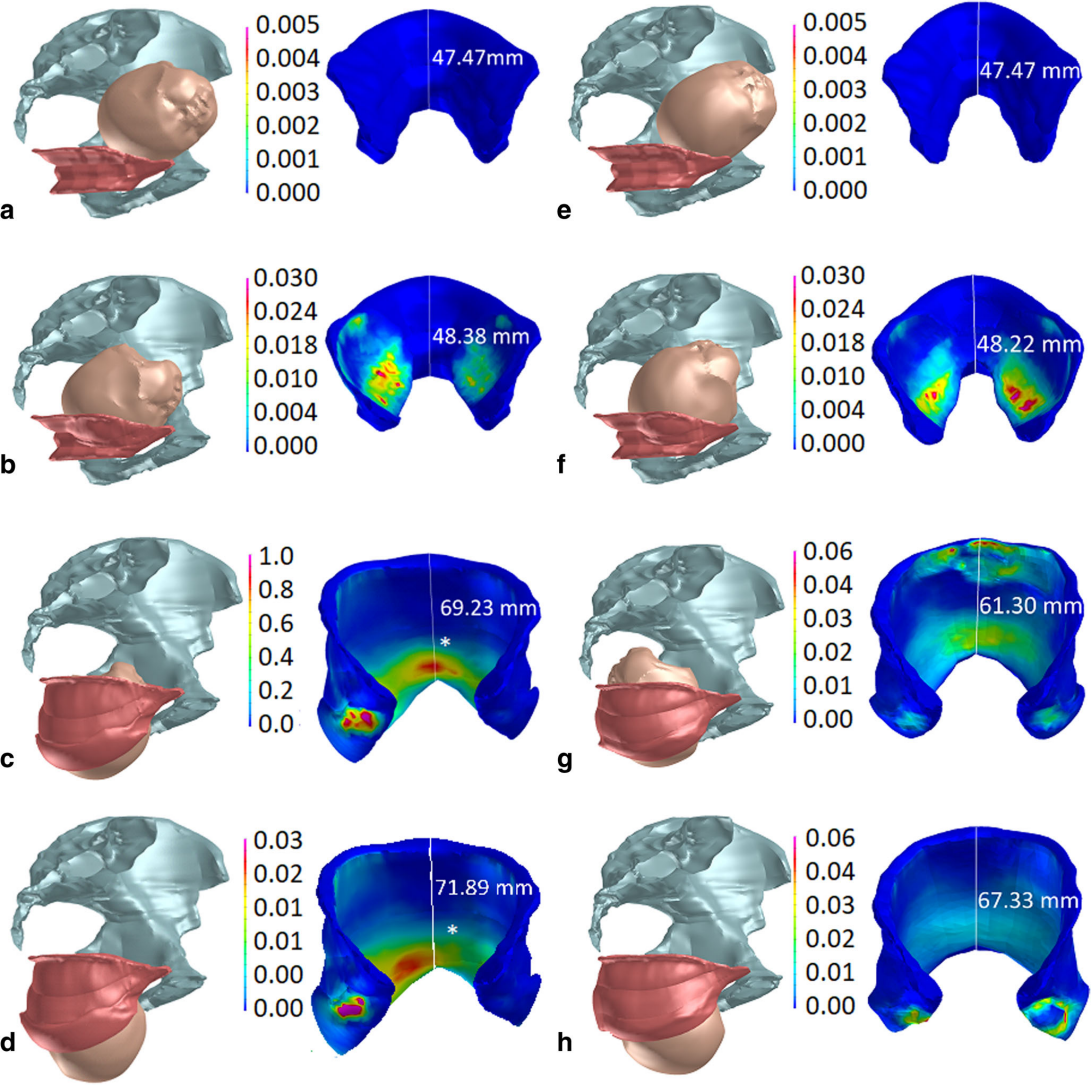
Color-coded view of the LAM areas demonstrating the von Mises stress distribution at various levels of OP fetal head descent. All stress values given in GPa. Muscle elongation in the caudal direction also depicted (mm). The right side view showing the actual position of the fetal head in relation to the pelvis. The frontal view showing the stress distribution in the whole LAM. **a** The leading point lies 1–2 cm above line 0 (station −2). At this level, stress changes in the ICm are not present. **b** The leading point is localized 3–4 cm below line 0 (*station +3*); the von Mises stress increases in the dorsolateral part of the PRm. **c** The leading point is placed 5–7 cm below line 0. The fetal forehead is in contact with the inferior margin of the symphysis pubis and head extension starts. The greatest stretch is induced in the PVm complex with the PRm originating from the dorsal surface of the pubic bone (marked with pink and red color). The most distal point on the PRm (*) loop is displaced 69.23 mm in the caudal direction as a result of distension by the fetal head. **d** The leading point is placed > 7 cm below line 0. The extension is going on. The distribution of stress in the PVm with the PRm originating from the dorsal surface decreases. The most distal point on the PRm (*) loop is displaced by a maximum of 71.89 mm in the caudal direction. **e**–**h** Comparison of the fetal head descent with OA

The calculated Ogden parameters, μ_1_, μ_2_, α_1_ and α_2_, computed for each pig’s LAM specimen are summarized in Table 1. Two members of the Ogden series were considered. The mean values ± standard deviations of the Ogden parameters were calculated: μ1 = 8.9 ± 8.2 GPa; μ2 = 21.6 ± 17.3 GPa; α1 = 0.1803 ± 0.1299; α2 = 15.112 ± 3.1704. The von Mises stress distribution in the LAM is shown in Table 2. For comparison, the table also contains the von Mises stress distribution in the LAM during vaginal delivery with respect to the cardinal movements of labor where the fetal head is in the OA position. The results are presented respecting the fetal head descent, considering the range from −1 up to +8. The fetal head initially distends the ICm portion of the LAM at station −1 and 0, respecting the OP and OA fetal head position. The mean stress values at this station were 1.15 MPa and 0.09 MPa, respectively. The PVm complex portion was distended at station +2 and +1 for the OP and OA head position. The mean stress values in these stations were 2.56 MPa and 0.01 MPa. Finally, the PRm portion was also distended at station +2 and + 1. The stress values were 120.28 MPa and 32.55 MPa, respectively. The maximal stress for the OP head position was found already at station +6 in the PRm muscle part. The mean value was 120.28 MPa. However, the maximum for the OA position was at station +8 in the PVm part. The value was 44.53 MPa. The color-coded versions of the von Mises stress distribution in the LAM during VD considering the LOP initial fetal head position is shown for four head positions (Fig. 3). In this figure, the MLA elongation in the caudal direction as a result of distention by the fetal head is also depicted. Considering both initial head positions, the MLA was elongated by nearly 1.5 times versus its initial resting position.

**Table 1:** Ogden parameters computed for each specimen

Sample no.	Material constants of the hyperelastic Ogden model			
	μ_1_ [MPa]	α_1_ [−]	μ_2_ [MPa]	α_2_ [−]
1	0.0074	0.0800	0.0000	5.9561
2	0.0043	0.0400	0.0000	6.3397
3	0.0016	0.2234	0.0011	19.300
4	0.0295	0.5006	0.0057	11.678
5	0.0019	0.0135	0.0022	9.0668
6	0.0001	0.0010	0.0136	10.156
7	0.0059	0.2094	0.0352	20.608
8	0.0046	0.2464	0.0229	13.405
9	0.0029	0.2267	0.0584	14.055
10	0.0027	0.2784	0.0516	14.167
11	0.0025	0.1328	0.0509	11.057
12	0.0019	0.1531	0.0228	15.995
13	0.0062	0.0932	0.0081	19.444
14	0.0010	0.0400	0.0212	20.034
15	0.0096	0.2571	0.0192	14.736
16	0.0029	0.1698	0.0108	15.058
17	0.0082	0.0803	0.0342	16.270
18	0.0270	0.1480	0.0333	16.947
19	0.0262	0.2640	0.0156	12.308
20	0.0172	0.4486	0.0248	15.667
Mean (± SD)	0.0089 (± 0.0082)	0.1803 (± 0.1299)	0.0216 (± 0.0173)	15.112 (± 3.1704)

Mean value (mean) and standard deviation (SD) summarized

**Table 2:** Relationship between fetal head descent and von Mises stress distribution (MPa) in selected LAM subdivisions

Head descent (cm)	von Mises stress (MPa)					
	Upper dorsal LAM portion (Iliococcygeus m.)		Left attachments, anteromedial LAM portion (pubovisceral and puborectal m.)		Distal posteromedial LAM portion (puborectal m.)	
	OP	OA	OP	OA	OP	OA
-1	0	0.09 ± 3.89	0	0	0	0
0	1.15 ± 3.33	0.13 ± 0.42	0	0	0	0
1	2.19 ± 14.21	2.09 ± 12.49	0	0.01 ± 0.01	0	10.19 ± 9.98
2	4.05 ± 16.31	2.52 ± 6.13	2.54 ± 6.78	0.67 ± 0.82	16. 96 ± 9.91	11.71 ± 10.32
3	5.08 ± 17.40	6.97 ± 7.73	5.43 ± 4.08	1.19 ± 1.53	25.90 ± 32.90	30.88 ± 30.41
4	8.66 ± 15.67	7.61 ± 10.89	7.30 ± 15.44	5.67 ± 6.09	52.29 ± 77.68	32.55 ± 20.51
5	9.86 ± 16.96	2.71 ± 3.38	16.14 ± 10.44	3.10 ± 2.68	80.83 ± 53.37	11.99 ± 4.13
6	16.80 ± 13.55	15.09 ± 19.83	95.50 ± 50.73	20.42 ± 15.79	120.28 ± 98.65	17.58 ± 8.99
7	3.90 ± 12.01	3.95 ± 10.464	65.73 ± 34.95	19.08 ± 20.89	42.66 ± 15.59	1.39 ± 1.21
8	1.73 ± 13.90	3.365 ± 5.146	35.90 ± 40.53	44.53 ± 34.95	12.36 ± 11.73	1.16 ± 0.34

Comparison of models considering the OP and OA fetal head position (mean value ± standard deviation). The head was simulated with a biparietal diameter of 93.7 mm, respecting the 50th percentile

## Discussion

Failure of the occiput to spontaneously rotate to the anterior position has been associated with increased maternal and neonatal morbidity. Its clinical significance has long been a subject of controversy among obstetricians. Despite several studies on this topic, the optimal method of decreasing the maternal pelvic floor and perineal trauma remains uncertain.

Our study provides original data on LAM behavior during the active second stage of labor in fetuses with a persistent OP position. It confirmed the assumption of direct dependence of the stress distribution in the LAM on the fetal head position. This work shows the significant stress increase for the case of the LOP position compared with the optimal OA position. The mechanism of VD of OP fetuses requires an increased distension of the LAM to accommodate a greater degree of downward descent of the fetal head until the forehead has completely passed the pubic bone. In the ICm, the stress values were comparable for both positions. Nevertheless, the von Mises stress generated in the PVm part was more than two times higher in the OP than OA position. In addition, the stress values in the PRm even increased 3.6 times. Nevertheless, the muscle elongation in the caudal direction was also comparable for both head positions.

**Fig. 4:**
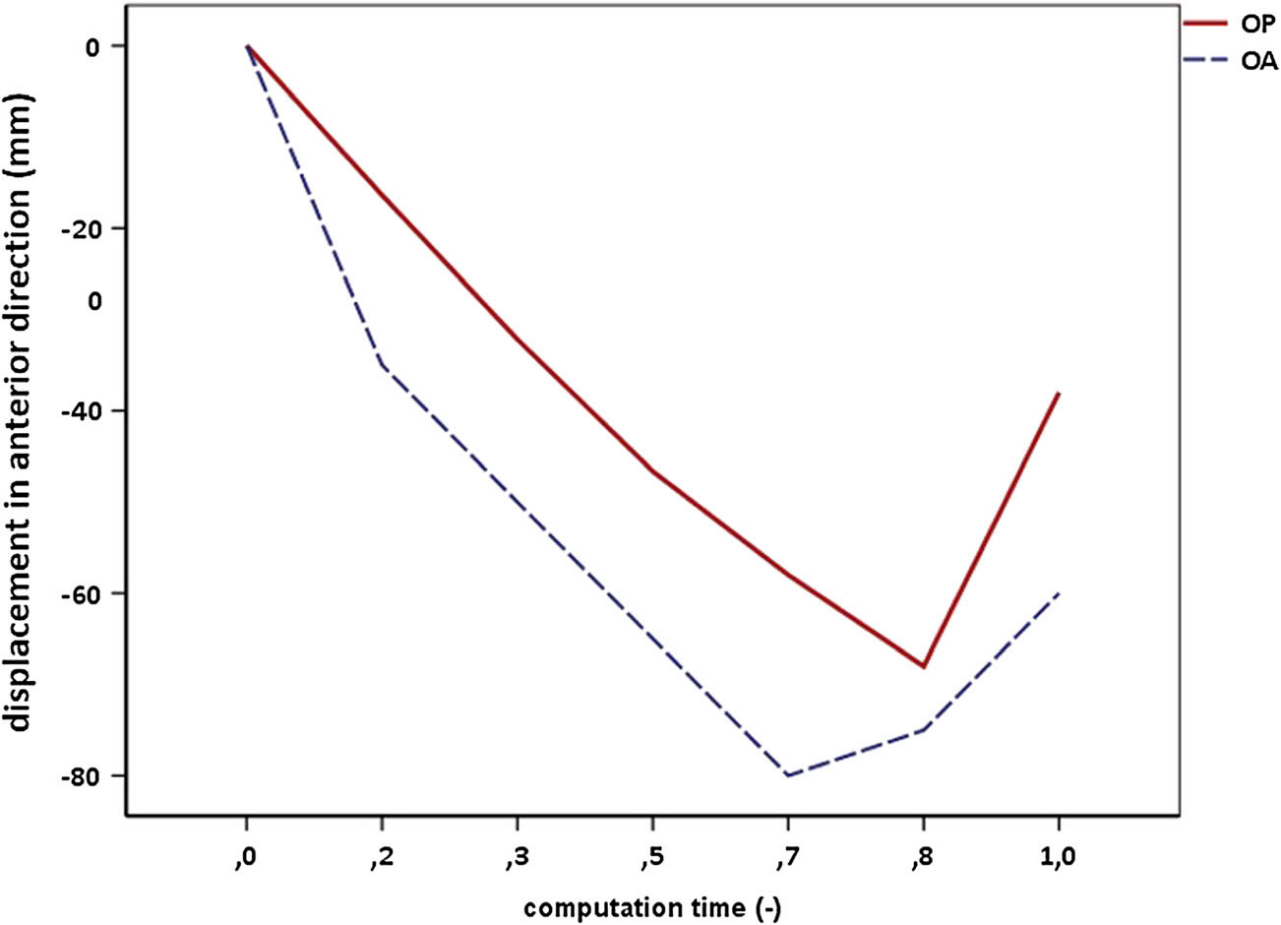
Comparison of biomechanical descriptions of the fetal head trajectories in the birth canal in the craniocaudal direction for the OP and OA positions. In OA the entire fetal head trajectory is given by the anatomical axis and follows the cardinal movements of labor. In vertex-presenting fetuses, head descent converts from downward at the pelvic inlet to horizontal at the midpelvis to upward at the outlet. In persistent OP, the head trajectory is different. OP fetuses maintain a less downward direction for a longer time with respect to the pubic bone from the pelvic inlet to midpelvis. After the ischial spine has been passed and the forehead has completely passed the pubic bone, flexion of the head becomes possible, and it abruptly twists up toward the outlet. After this upward change, the path of OP fetuses is likely to become similar to that of OA fetuses in the later phase of the second stage

In the presented model, the largest LAM stretch ratio was 1.5 in the inferior direction. Compared with the range of 1.62–3.76, based on a statistical study of 227 women [28], the LAM seems to be slightly stiffer. This could be caused by the pig muscle specimens used for material constant identification. Nevertheless, the model was well validated comparing the results of the OA head position with the published data. The maximal mean stress in the ICm was 15.09 MPa, which corresponds to the results in the literature [16, 29]. The maximal stress values in the PVm complex were generated during fetal head extension. The obtained value of 44.53 MPa is also comparable to the literature [16]. Finally, the maximum value in the posteromedial inner area of the PRm was 32.55 MPa at station +4, corresponding with data from [13]. The authors were unable to find any literature describing the simulation of the OP FEM model; thus, we were unable to validate these results. Nevertheless, the FEM model was validated for the LOA head position. Therefore, the outputs for the OP position can also be considered as correct. This increase in LAM stress distribution in OP may be associated with a variety of factors. Incomplete flexion of the fetal head increases the presenting diameter and alters the mechanics of labor. Fetuses with OA follow the curved path of the birth canal, whereas fetuses with OP show a different head trajectory (Fig. 4). This different trajectory has been sonographically witnessed among fetuses with persistent OP positions in the active second stage [30]. This confirms our observations that the mechanism of VD in OP fetuses requires an increased LAM distension to accommodate a greater degree of downward fetal head descent.

Concerning the material modeling, the presented model has advantages and also some limitations. The first limitation of this model is that the parameters of the pig LAM were measured instead of human tissues. The porcine LAM has the same structure, function and attachments as the human. Nevertheless, porcine bodies have a different anatomy. They move on four legs; moreover, their fetuses have different weights and sizes in the ratio of female parameters compared with the human body. Therefore, the external forces acting on the pig LAM are dissimilar to those of humans, and the mechanical properties could be slightly different. The second limitation is that we did not measure in vivo muscles. The results of measurements of mechanical properties could be influenced by dehydration of the tissue. However, this potential source of bias was minimalized by the short duration of the experiment (< 10 min) and by spraying the tissue with a saline solution. The third limitation is that the muscle samples were mechanically dissected from the porcine pelvis and thus the tissue could be slightly deformed involving some volume changes. The next limitation is that the measurement and model were simplified. The LAM is an anisotropic biological tissue. Therefore, the use of uniaxial testing together with the isotropic constitutive model is not fully sufficient to describe their mechanical response. For our purpose, this analysis is acceptable. Nevertheless, to characterize the anisotropic properties, a cross-fiber experiment should be performed. Another limitation is that the head was driven at a given trajectory with a homogeneous progression. Linear progress is used as a simplification of real labor in our numerical simulation. We plan to consider more realistic motion of the fetal head (progress and standstill) in our future studies.

Unlike other studies in which the head model is oversimplified as a sphere, we used the geometry of an actual neonatal head based on MRI. The effects of fetal head molding were not considered. However, the use of a deformable fetal head leads to an almost 20% reduction of the reaction forces on the pelvic floor muscles compared with a rigid head [31]. Nevertheless, to the authors' knowledge, there is no data set in the literature describing head molding during VD considering the OP head position.

In the future, this model will be improved in a few steps: a biaxial tensile test will be performed to obtain the material constants to allow the simulation of non-homogeneous behavior of muscle tissue; a molding head for the LOA position will be used.

Our results have clearly shown that a persistent OP position is a significant risk factor for LAM stress injury. Recognition of this greater susceptibility to severe birth-related LAM trauma at OP delivery should help reduce its occurrence.

## Abbreviations

MRI: Magnetic resonance imaging
LAM: Levator ani muscle
VD: Vaginal delivery
PFD: Pelvic floor dysfunction
OP: Occiput posterior
OIm: Obturator internus muscle
ICm: Iliococcygeal muscle
PRm: Puborectal muscle
PVm: Pubovisceral muscle
FEM: Finite element mesh
ICs: Initial conditions
BCs: Boundary conditions
ATML: Arcus tendineus musculi levatoris ani
OA: Occiput anterior
LOP: Left occiput posterior
AR: Aspect ratio
GPa: Gigapascal
MPa: Megapascal
